# TLIF Online Videos for Patient Education—Evaluation of Comprehensiveness, Quality, and Reliability

**DOI:** 10.3390/ijerph20054626

**Published:** 2023-03-06

**Authors:** Stephan Heisinger, Dominikus Huber, Michael P. Matzner, Helmut Hiertz, Lukas Peter Lampe, Jakob Zagata, Stefan Aspalter, Christian Radl, Wolfgang Senker, Georg Mair, Josef G. Grohs

**Affiliations:** 1 Department of Orthopedics and Trauma Surgery, Medical University of Vienna, 1090 Vienna, Austria; 2 Division of Oncology, Department of Medicine I, Medical University of Vienna, 1090 Vienna, Austria; 3 Division of Neurosurgery, Medical Health Centre Bad Vigaun, 5424 Bad Vigaun, Austria; 4 Department of Orthopaedics and Trauma Surgery, St. Josef-Hospital, Ruhr-University Bochum, 44791 Bochum, Germany; 5 Department of Neurosurgery, Klinik Landstraße, 1030 Vienna, Austria; 6 Department of Neurosurgery, Kepler University Hospital, 4020 Linz, Austria; 7 Department of Orthopedics and Traumatology, Hanusch Hospital of OEGK, 1140 Vienna, Austria

**Keywords:** spine, digital health, lumbar region, TLIF, educational film and video

## Abstract

Over the last few decades, the number of lumbar interbody fusion surgeries performed has been constantly increasing, with transforaminal lumbar interbody fusion (TLIF) being one of the most common surgical techniques. Due to easy accessibility, patients frequently use YouTube to obtain information on health-related issues. Consequently, online video platforms may be a valuable tool for patient education. The aim of this study was to assess the quality, reliability, and comprehensiveness of online videos on TLIF. We screened 180 videos on YouTube, yielding a total of 30 videos that met the inclusion criteria. These videos were evaluated using Global Quality Scale, DISCERN reliability tool, and JAMA Benchmark Score, and assessed in regard to their comprehensiveness and coverage of relevant aspects. At the time of rating, the videos had between 9188 and 1,530,408 views and between 0 and 3344 likes. The median rater assessment for all videos was “moderate quality”. GQS and subjective grades showed a moderate to strong statistically significant association with views and likes. Considering this association of GQS and subjective grade with views and likes, these criteria could be used by laypersons to identify good-quality content. Nevertheless, there is an urgent need for peer-reviewed content that covers all of the relevant aspects.

## 1. Introduction

Transforaminal lumbar interbody fusion (TLIF) surgery is an established procedure to treat a wide range of lumbar spine pathologies, e.g., degenerative pathologies, trauma, and infection [1,2]. Moreover, the volume of lumbar fusion surgery and consequently revision surgery is constantly increasing, thus imposing a significant socioeconomic burden [3,4]. Martin et al. found that the volume of elective lumbar fusion increased by 62.3%, from 122,679 cases in 2004 to 199,140 in 2015, in the United States of America [5]. As reviewed by Kaustubh et al., this may be attributed to a variety of reasons such as the aging of the population, patient expectations, improved anesthetic and perioperative management, as well as new surgical techniques leading to faster recovery and more favorable outcomes [4,6,7,8].

Over the last decade, technological advancements and, more recently, the COVID-19 pandemic have continuously led to the establishment and further development of telemedicine and internet-based patient education [4]. As we have reviewed previously, numerous authors found that the majority of the North American population with access to the internet uses it to obtain information on health-related issues, and that the internet has become one of the most important sources of health education [9,10,11,12,13]. Moreover, YouTube (Alphabet, Mountain View, CA, USA) has become one of the most influential websites in regard to health education and information, with more than 1 billion visitors monthly and almost three-quarters of the U.S. population using it [10,14,15]. However, the content does not undergo peer-review before being uploaded; consequently, there is an immanent risk of obtaining wrong or misleading information [10,16,17,18]. Considering the increasing number of patients acquiring health-related information from YouTube (Alphabet, Mountain View, CA, USA), the company is taking actions to address this relevant issue [19].

While various authors have assessed online information on spine surgical procedures and even lumbar fusion, to our knowledge, there is to date no study reviewing the online media content on TLIF surgery in particular [4,20,21,22,23]. Given the variety of lumbar interbody fusion techniques such as TLIF, XLIF, OLIF, ALIF, and PLIF, and TLIF surgery being one of the most common surgical procedures for interbody fusion, the online media content on TLIF surgery needs to be carefully evaluated in regard to its quality, reliability, and even more importantly its comprehensiveness. Moreover, high-quality content strongly supports patients in making an informed decision as the videos can be re-watched, paused, etc., and may be watched in a less stressful environment, thereby enhancing adequate information uptake and processing. Eastwood et al. were able to show that extensive multidisciplinary preoperative patient education resulted in significantly better postoperative outcomes, thereby highlighting the crucial role of patient education in terms of successful surgical interventions [24]. Moreover, we strongly believe that freely accessible online content that is comprehensive and of high quality would generally be a valuable tool to further improve patient education and subsequently postoperative outcomes. As observed by Phan et al., the majority of the most commonly accessed online patient education material pertaining to surgical treatments of the spine exceeded the readability limits recommended by the American Medical Association and the National Institutes of Health, consequently suggesting that patients would not be able to comprehend the provided content [25]. This further highlights the need for high-quality yet also easily comprehensive online content.

Therefore, we aim to evaluate the comprehensiveness, reliability, and quality of online videos on TLIF surgery using established scoring tools for online media. Moreover, we aim to evaluate the suitability and reproducibility of these established online media scoring tools. Furthermore, we hypothesize that the currently available scoring tools are limited in regard to their ability to provide an objective and reproducible assessment.

## 2. Materials and Methods

Analogous to previous studies that evaluated online videos, this study’s design was purely descriptive, and in a similar fashion we used 3 search items: “TLIF”, “TLIF surgery” and “Transforaminal Interbody Fusion” on YouTube (www.youtube.com, accessed on 25 October 2021) [10,26]. The results were sorted by view count, and the 60 most-viewed videos from each search were evaluated further [26]. Consequently, 180 videos were screened, and off-topic videos, duplicates, videos with a language other than English, and otherwise inadequate videos were excluded [26]. Thirteen videos were excluded as they merely contained patient reported outcome reports and consequently could be considered off-topic in regard to our study design; another five videos were excluded due to the use of a language other than English. Furthermore, we excluded 11 videos that either lacked any verbal or written description or explanation, or merely depicted the surgical procedure. We further excluded two promotional videos and another two videos which focused on the comparison of different procedures. Another 18 videos were excluded because they covered surgical procedures other than TLIF surgery. Furthermore, all duplicates were eliminated. Finally, the screening and selection process resulted in 30 videos that met the inclusion criteria. We recorded the title of the video, universal resource locator, number of total views, number of likes, number of dislikes, duration in seconds, and source of the videos. The sources were classified as MD (Medical Doctor), HC institution (healthcare institution), patient, and HC company (health care company).

The resulting 30 videos were further assessed by 8 observers—4 neurosurgeons and 4 orthopedic surgeons (R1-R8). All of the observers were trained and experienced spine surgeons and were fluent in spoken and written English. Analogous to previous studies, we used 3 acknowledged scoring and grading systems to assess the media content: Global Quality Scale (GQS), modified DISCERN tool, and the JAMA benchmark score [26,27,28]. Additionally, the observers rated the videos on a subjective basis, as well as in regard to technical sound and video quality, using a scale ranging from 1 to 5 (1 = excellent to 5 = insufficient). Observers determined whether advertisements were present (yes/no). In order to determine whether the videos could be suited for patient education, the observers evaluated the following 4 questions in a binary yes/no manner: “Is it easily understandable for laypersons?”, Are the risks/complications discussed?”, “Is the procedure described adequately?”, and “Is the rehabilitation described adequately?”

## 3. Statistical Analysis

Data were stored and processed for further analysis in MS Excel (Microsoft Corporation, 2018. Microsoft Excel, available online: https://office.microsoft.com/excel (accessed on 6 June 2020). Statistical analysis was carried out in GraphPad Prism (GraphPad Prism version 9.1.0 for Windows, GraphPad Software, San Diego, CA, USA, available online: www.graphpad.com accessed on 6 June 2020) and SPSS (IBM Corp. Released 2020. IBM SPSS Statistics for Windows, Version 27.0. Armonk, NY, USA: IBM Corp.).

Descriptive analysis included mean (metric variables) and percentiles (scores). Hypothesis testing (for difference in medians) employed Mann–Whitney U tests, association was estimated via Spearman’s rho, and rater agreement using Fleiss’ Kappa. Difference in distribution was tested via modified Chi-Square tests for multiple variables.

An alpha of 0.05 was assumed to constitute statistical significance. Where appropriate, confidence intervals are reported, also using alpha = 0.05. In all cases, two-sided testing was performed.

## 4. Results

Eight surgeons (four orthopedic surgeons, four neuro-surgeons) rated a total of 30 YouTube videos. At the time of rating, the videos had between 9188 and 1,530,408 views and between 0 and 3344 likes (Table 1).

**Figure 1:**
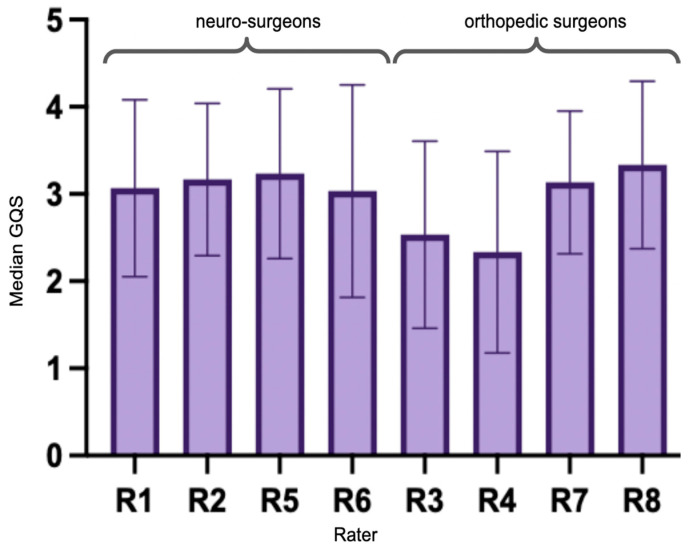
Median GQS for each rater (R1-8). R1, R2, R5, and R6 were neurosurgeons. R3, R4, R7, and R8 were orthopedic surgeons. The latter assigned significantly lower grades to the videos.

The median rater assessment for all videos was “moderate quality” (GQS = 3). Poor quality (GQS = 1) was rated in 8.3%, “generally poor” (GQS = 2) in 26.7%, “moderate quality” (GQS = 3) in 35.0%, “good quality” (GQS = 4) in 27.1%, and “excellent quality” (GQS = 5) in 9.2% of cases. There was a significant difference between raters regarding their GQS (Kruskal–Wallis test *p* = 0.003) in that rater 4 (R4) assigned significantly lower scores than R2 (Dunn’s correction for multiple comparisons *p* = 0.038) and R5 (*p* = 0.015) as well as R8 (*p* = 0.012). In total, the neurosurgeons rated the videos higher than the orthopedic surgeons (Mann–Whitney U test *p* = 0.047, Figure 1). There was slight rater agreement on GQS, indicated by Fleiss’ Kappa = 0.13 (*p* < 0.05).

**Figure 2:**
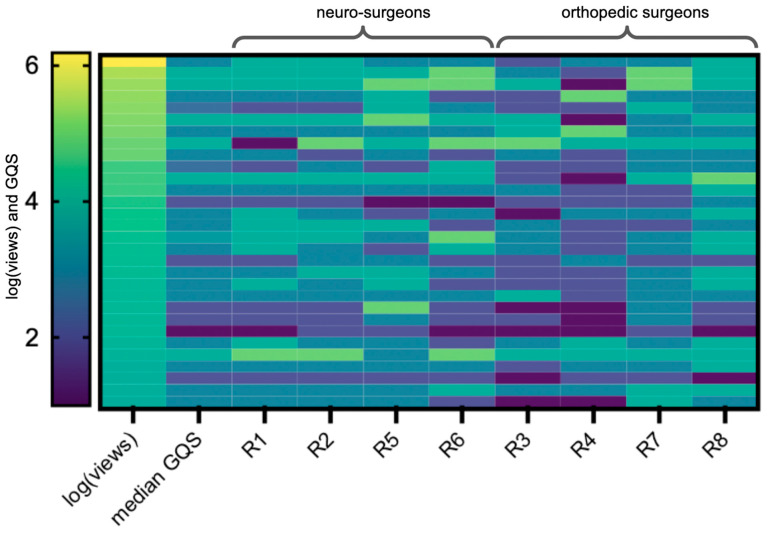
GQS scores for each video (rows) from all raters (column 3 to 10) and their median (column 2) were ordered by the logarithm of the respective views of each video (column 1). The darker the color, the fewer views and the worse the rating.

There was a strong and highly significant correlation between median GQS and the number of views (Spearman’s rho = 0.48, *p* = 0.007). Only the individual rating of rater 6 (Spearman’s rho = 0.48, *p* = 0.007) and rater 7 (Spearman’s rho = 0.49, *p* = 0.006) showed a significant and strong association with views (Figure 2). Median GQS also showed correlation with the number of likes, and again only the scores assigned by rater 6 were statistically significantly associated (Spearman’s rho = 0.50, *p* = 0.005).

Only raters 6 and 7 showed a statistically significant assignment of scores regarding views. Overall, there was a strong correlation between median GQS score and views, rendering this YouTube characteristic possibly an effective tool when judging the quality of a video.

**Figure 3:**
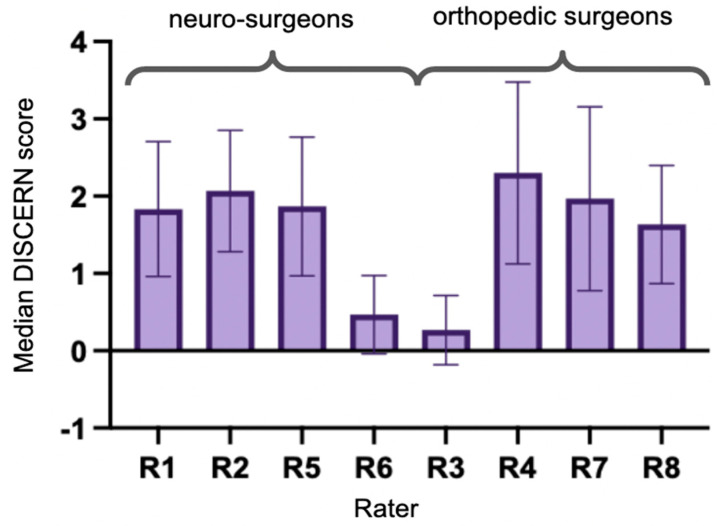
Median DISCERN Scores for each rater (R1-8). R6 and R3 assigned significantly lower DISCERN scores than the others.

The median DISCERN score was 2. In total, 19.7% were rated with a score of 0, 30.0% with a score of 1, 32.9% with a score of 2, 13.8% with a score of 3, 2.9% with a score of 4, and only 1.3% with a score of 5. Raters R6 (neurosurgeon) and R3 (orthopedic surgeon) differed significantly in their rating from the others (Kruskal–Wallis test *p* < 0.001 and multiple comparisons with these two raters corrected via Dunn’s method all *p* < 0.001, Figure 3). A Mann–Whitney U test showed no significant difference between neurosurgeons and orthopedic surgeons (*p* = 0.49). Rater agreement on DISCERN score was poor (Fleiss’ Kappa = −0.08). No association could be found between views or likes and the overall and individually assigned DISCERN scores.

**Figure 4:**
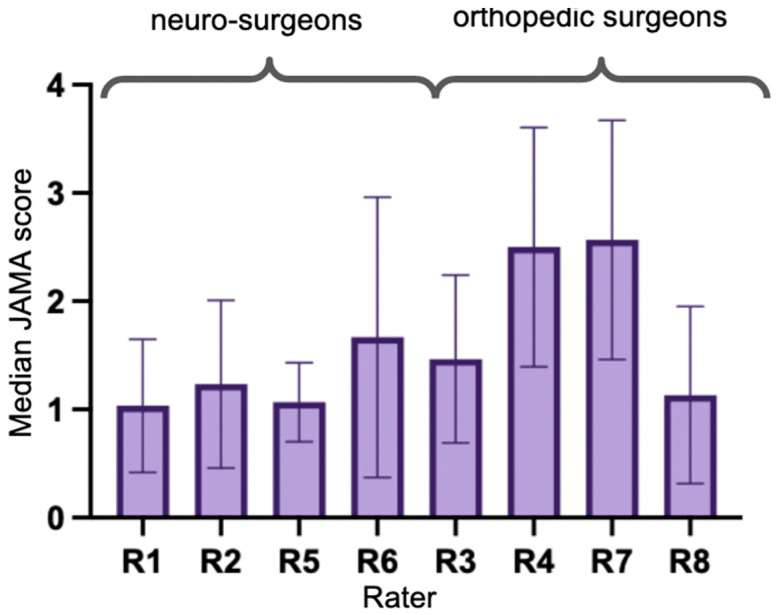
Median JAMA scores for each rater (R1-8). R4 and R7 assigned significantly higher JAMA scores than the others. Orthopedic surgeons assigned significantly higher JAMA scores in total.

The median JAMA score assigned to the videos was 1. Overall, 10.0% of scores were 0, 48.3% were 1, 22.5% were 2, 11.7% were 3, and 7.5% were 4. All neurosurgeons and one orthopedic surgeon assigned median scores of 1, while 2 orthopedic surgeons assigned median scores of 3 and 4 (Figure 4). These raters (R4 and R7) differed significantly from the other raters (Kruskal–Wallis test *p* < 0.001, Dunn’s corrected multiple comparisons *p* < 0.001 for combinations with these two raters). Neurosurgeons assigned significantly lower JAMA scores than orthopedic surgeons (Mann–Whitney U test *p* < 0.001). Rater agreement on JAMA score was poor (Fleiss’ Kappa = 0.03). No associations were found between views or likes and the overall and individually assigned JAMA scores.

When assessing the content based on the aforementioned questions, there was very poor rater agreement (Fleiss’ Kappa for all questions < 0). Data is presented for each rater individually and the median of all raters (Table 2). In the median, 80% of videos described the procedure adequately, and 55% were easily understandable by laypersons. However, only 10% of videos were perceived to describe rehabilitation and 83% to describe risks/complications adequately. There was no statistically significant correlation between the answers of individuals or between surgical specialties.

**Figure 5:**
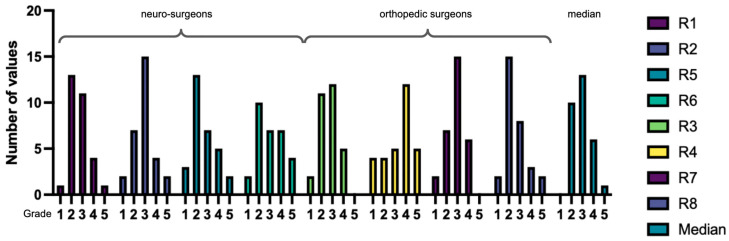
Histograms of subjective overall grades in the median (column 1) and raters 1 to 8 (columns 2 to 9).

Subjective grades (1 = excellent to 5 = insufficient) were given to each video by each rater for overall, video, and sound quality. Overall quality grades had a median of 3 for both surgical specialties, combined with a 95% confidence interval of (2;3). This grade was weakly correlated with both views (Spearman’s rho = −0.39, *p* = 0.036) and likes (Spearman’s rho = −0.39, *p* = 0.032) in its median value, and for rater 2 (rho = −0.43, *p* = 0.017), rater 4 (rho = −0.41, *p* = 0.022), and rater 5 (rho = −0.44, *p* = 0.023) individually. No significant difference between neurosurgeons and orthopedic surgeons could be detected (Mann–Whitney U test *p* = 0.21) but the histograms of orthopedic surgeons appear more skewed to the right, indicating a higher number of worse grades (Figure 5).

Overall, 7.5% of videos received a grade of 1, 33.3% grade 2, 33.3% grade 3, 19.2% grade 4, and 6.7% grade 5. Fleiss’ Kappa for subjective overall grade was 0.18, indicating slight agreement between raters. Spearman’s rho indicated a very strong, highly significant correlation with GQS (rho = −0.90, *p* < 0.001).

Median sound and video quality grades were both 2 with a 95% confidence interval of (1,2). In total, 41.3% of videos were graded with “excellent” video quality, 32.9% with a grade of 2, 15.4% with grade 3, 7.5% with grade 4, and 2.9% with grade 5. In total, 45.8% were graded with “excellent” sound quality, 30.0% with grade 2, 11.3% with grade 3, 6.7% with grade 4 and 6.3% with grade 5. Both video (Spearman’s rho = 0.62, *p* < 0.001) and sound (Spearman’s rho = 0.49, *p* < 0.001) were strongly and highly significantly correlated with subjective overall grade. When compared to GQS, both video (Spearman’s rho = −0.58, *p* < 0.001) and sound (Spearman’s rho = −0.44, *p* = 0.015) showed a strong and statistically significant association with this form of scoring.

In summary, both GQS and subjective grades showed a moderate to strong, statistically significant association with views and likes. These two criteria could be used by laypersons to judge the quality of the video content. Subjective overall grade, and to a slightly lesser extent GQS, showed strong correlations with video and sound quality.

## 5. Discussion

Nowadays, patients do not merely rely on their doctors to obtain information on their medical conditions and, more specifically, invasive procedures that they may be offered to treat their conditions. Although easily and quickly accessible, the reliability and validity of internet-based sources remain controversial topics and major concerns especially for patients without any medical education [10,12,13,26,29].

Considering the constantly increasing volume of lumbar fusion surgeries performed during the preceding decades as well as concomitant technological advancements such as smartphones, and the popularity of YouTube and other social media platforms, it is hardly surprising that patients use these new tools to acquire health-related information [5,10,14,15]. Nevertheless, the quality and reliability of the available online videos are highly variable due to a lack of a peer-review process. However, the company is starting to take actions to address this relevant issue [19].

Various authors have investigated the quality and reliability of YouTube videos on lumbar fusion or arthroplasty in general and, more specifically, for anterior lumbar interbody fusion (ALIF) or lateral lumbar fusion (LLIF); but to our knowledge, there are no studies that evaluated online videos on TLIF surgery [4,20,21,22,23]. Given that TLIF surgery is a very common surgical procedure, it is crucial to evaluate the available YouTube videos on TLIF in regard to quality, reliability, and comprehensiveness.

Unlike other studies, we did not limit our scoring system to merely one or two scores. We used three acknowledged scoring and grading systems to assess the media content: Global Quality Scale (GQS), modified DISCERN tool, and the JAMA benchmark score [26,27,28]. Furthermore, the videos were rated on a subjective basis as well as in regard to technical sound and video quality, and it was determined whether advertisements were present. Additionally, the videos were evaluated in regard to their suitability for patient education by the following four questions: “Is it easily understandable for laypersons?”, “Are the risks/complications discussed?”, “Is the procedure described adequately?”, and “Is the rehabilitation described adequately?” All of these additional aspects that we considered and evaluated have given us an immense amount of information and profound insight into the current quality, reliability, and comprehensiveness of the available media. Another advantage of this study is the number of observers—four orthopedic surgeons and four neurosurgeons evaluated the videos—thereby providing a more objective overall assessment. We chose to involve a fairly large number of observers compared to other studies to compensate for any outliers. For example, R3 and R6 differed significantly in their ratings from the others. If we had been limited to these two raters, we would have come to completely different conclusions, which consequently highlights the importance of a larger number of raters or observers. Interestingly, we found that orthopedic surgeons assigned significantly lower GQS and JAMA score ratings to the videos, which may be a coincidence or may also reflect the specialties’ different approaches to this topic.

We found a strong and highly significant correlation between median GQS and the number of views, which may indicate good quality in this specific inquiry. However, we also observed that GQS, as well as subjective grading, is strongly biased by technical sound and video quality. These finding consequently suggest that there is a need for more objective scoring that separately displays the information quality and the technical qualities more distinctively.

In contrast to our study, various authors have found the information on YouTube on lumbar fusion to be poor quality, while we determined the median rater assessment for the videos on TLIF surgery to be “moderate quality” [4,20,22]. Interestingly our literature review also yielded studies that focused on lateral lumbar interbody fusion, which found the according media content on YouTube to be of moderate quality [21,23].

We observed a slight rater agreement on GQS; however, we also found that neurosurgeons rated the videos significantly higher than the orthopedic surgeons.

Unlike other studies that evaluated online videos, we found that the median GQS also showed a correlation with the number of likes [26].

Regarding the DISCERN tool, we found rater agreement was poor, and no association could be found between views or likes and the overall and individually assigned scores.

Concerning the JAMA score, which evaluates aspects such as authorship, attribution, disclosures, and currency, we observed that the neurosurgeons rated the videos lower than the orthopedic surgeons. Analogous to our findings with the DISCERN tool, the rater agreement was poor and no association could be found between views or likes.

These findings, especially in regard to rater agreement when using the different scoring systems, are highly interesting and moreover relevant for future studies in this field. The poor rater agreement with DISCERN reliability scores and JAMA scores appears especially crucial considering that various studies rely on either of these scores as the sole assessment tool. We consequently hypothesize that the outcome of these online media evaluation studies greatly depends on the chosen scores, which we found to show rather limited rater agreement. Consequently, we suggest that the GQS is included in future studies in this field, and we should consider establishing a new scoring system that offers a more objective assessment. We therefore conclude that the scores that are currently available to assess online media content are in fact not entirely suited for this task, and the according results have to be interpreted in regard to this limited capacity to assess the content in a reproducible manner. Moreover, the lack of suited assessment tools is one of the main limitations of this study. Further limitations of this study are that all of the applied scoring systems are based on the observer’s subjective judgement, and this study represents a snapshot in time, which may be controversial due to the dynamic structure of YouTube and the advances in online patient education [26]. Additionally, we only included videos in English, and search results may vary due to demographic differences [26].

Based on the content-related questions, we found that a median of 80% of videos described the procedure adequately, and 55% were easily understandable. However, only 10% of videos described rehabilitation. This indicates that there is a strong need for content that also addresses the aspect of the rehabilitation process. Furthermore, we observed that potential risks and complications were discussed in only 10% of the videos, which we found to be a major pitfall, and this crucially limits the usability of these videos in regard to patient education.

We observed that the majority of videos were graded as good or excellent in regard to their technical sound and video quality, and they showed a strong and statistically significant association with subjective overall grading and GQS.

## 6. Conclusions

Overall, we have determined the available YouTube videos on TLIF surgery to be of moderate quality based on their GQS scoring, which, as well as overall subjective grading, showed a strong association with likes and views. In this specific case, a layperson may use the number of views or likes to identify good-quality content. However, this association seems to be topic-specific and rather coincidental. Furthermore, we found a strong correlation between technical video and sound quality with GQS and overall subjective grading, which may, in this specific case, be used to identify high-quality content; however, it also indicates that GQS may be relevantly biased by technical video and sound quality. This finding consequently emphasizes the need for a more objective grading system that potentially displays the technical quality and information quality separately. Considering the low inter-rater agreement with JAMA score and DISCERN reliability tool, this has to be taken into account for the design of future studies especially if only one or two observers rate the content. Therefore, we advocate for the use of the GQS, which at least showed slight rater agreement.

Moreover, we found that the majority of videos do not address the aspect of rehabilitation nor the potential risks and complications, which is crucial in our opinion. Consequently, there is a need for more high-quality videos that also cover these aspects. Furthermore, we would like to highlight the importance of providing high-quality content that is easily comprehensible for the average patient.

In conclusion, we advocate for the establishment of a more suited assessment tool for online media content. In the specific case of TLIF surgery, there is a relevant need for content that also addresses the rehabilitation process in particular.

## Figures and Tables

**Table 1:** Descriptive statistics of views and likes of all 30 rated videos with percentiles and 95% confidence intervals of the median.

Variable	Views	Likes
Minimum	9188	0.000
25% Percentile	16,783	28.75
Median	30,997	78.00
75% Percentile	113,654	296.3
Maximum	1,530,408	3344
Range	1,521,220	3300
95% CI of the median		
Lower confidence limit	17,535	36
Upper confidence limit	55,873	159

**Table 2:** Questions regarding quality/content and percent of videos that each rater assigned the answer “true” to.

Question	R1	R2	R5	R6	R3	R4	R7	R8	Median
Is it easily understandable for laypersons?	80.00%	70.00%	46.67%	23.33%	53.33%	20.00%	66.67%	80.00%	55.00%
Are the risks/complications discussed?	10.00%	10.00%	10.00%	23.33%	20.00%	26.67%	23.33%	10.00%	8.33%
Is the procedure described adequately?	80.00%	80.00%	50.00%	76.67%	60.00%	60.00%	93.33%	76.67%	80.00%
Is the rehabilitation described adequately?	13.33%	13.33%	10.00%	13.33%	6.67%	6.67%	23.33%	13.33%	10.00%

## Data Availability

The data presented in this study are available on request from the corresponding author.

## References

[1] Mobbs R.J., Phan K., Malham G., Seex K., Rao P.J. (2015). Lumbar Interbody Fusion: Techniques, Indications and Comparison of Interbody Fusion Options Including PLIF, TLIF, MI-TLIF, OLIF/ATP, LLIF and ALIF. J. Spine Surg. Hong Kong.

[2] Resnick D.K., Choudhri T.F., Dailey A.T., Groff M.W., Khoo L., Matz P.G., Mummaneni P., Watters W.C., Wang J., Walters B.C. (2005). Guidelines for the Performance of Fusion Procedures for Degenerative Disease of the Lumbar Spine. Part 7: Intractable Low-Back Pain without Stenosis or Spondylolisthesis. J. Neurosurg. Spine.

[3] Saifi C., Cazzulino A., Laratta J., Save A.V., Shillingford J.N., Louie P.K., Pugely A.J., Arlet V. (2019). Utilization and Economic Impact of Posterolateral Fusion and Posterior/Transforaminal Lumbar Interbody Fusion Surgeries in the United States. Glob. Spine J..

[4] Ahuja K., Aggarwal P., Sareen J.R., Mohindru S., Kandwal P. (2021). Comprehensiveness and Reliability of YouTube as an Information Portal for Lumbar Spinal Fusion: A Systematic Review of Video Content. Int. J. Spine Surg..

[5] Martin B.I., Mirza S.K., Spina N., Spiker W.R., Lawrence B., Brodke D.S. (2019). Trends in Lumbar Fusion Procedure Rates and Associated Hospital Costs for Degenerative Spinal Diseases in the United States, 2004 to 2015. Spine.

[6] Kai-Hong Chan A., Choy W., Miller C.A., Robinson L.C., Mummaneni P.V. (2019). A Novel Technique for Awake, Minimally Invasive Transforaminal Lumbar Interbody Fusion: Technical Note. Neurosurg. Focus.

[7] Pendharkar A.V., Shahin M.N., Ho A.L., Sussman E.S., Purger D.A., Veeravagu A., Ratliff J.K., Desai A.M. (2018). Outpatient Spine Surgery: Defining the Outcomes, Value, and Barriers to Implementation. Neurosurg. Focus.

[8] Chin K.R., Coombs A.V., Seale J.A. (2015). Feasibility and Patient-Reported Outcomes After Outpatient Single-Level Instrumented Posterior Lumbar Interbody Fusion in a Surgery Center: Preliminary Results in 16 Patients. Spine.

[9] Heisinger S., Huber D., Matzner M.P., Hasenoehrl T., Palma S., Sternik J., Trost C., Treiber M., Crevenna R., Grohs J.G. (2021). Online Videos as a Source of Physiotherapy Exercise Tutorials for Patients with Lumbar Disc Herniation-A Quality Assessment. Int. J. Environ. Res. Public. Health.

[10] Springer B., Bechler U., Koller U., Windhager R., Waldstein W. (2020). Online Videos Provide Poor Information Quality, Reliability, and Accuracy Regarding Rehabilitation and Return to Sport After Anterior Cruciate Ligament Reconstruction. Arthrosc. J. Arthrosc. Relat. Surg..

[11] Diaz J.A., Griffith R.A., Ng J.J., Reinert S.E., Friedmann P.D., Moulton A.W. (2002). Patients’ Use of the Internet for Medical Information. J. Gen. Intern. Med..

[12] Fox S., Rainie L. (2002). E-Patients and the Online Health Care Revolution. Physician Exec..

[13] Starman J.S., Gettys F.K., Capo J.A., Fleischli J.E., Norton H.J., Karunakar M.A. (2010). Quality and Content of Internet-Based Information for Ten Common Orthopaedic Sports Medicine Diagnoses. J. Bone Joint Surg. Am..

[14] MacLeod M.G., Hoppe D.J., Simunovic N., Bhandari M., Philippon M.J., Ayeni O.R. (2015). YouTube As an Information Source for Femoroacetabular Impingement: A Systematic Review of Video Content. Arthrosc. J. Arthrosc. Relat. Surg..

[15] Pew Research Centre Social Media Usage: 2005–2015. https://www.pewresearch.org/internet/2015/10/08/social-networking-usage-2005-2015/.

[16] Madathil K.C., Rivera-Rodriguez A.J., Greenstein J.S., Gramopadhye A.K. (2015). Healthcare Information on YouTube: A Systematic Review. Health Inform. J..

[17] Lewis S.P., Heath N.L., Sornberger M.J., Arbuthnott A.E. (2012). Helpful or Harmful? An Examination of Viewers’ Responses to Nonsuicidal Self-Injury Videos on YouTube. J. Adolesc. Health.

[18] Dubey D., Amritphale A., Sawhney A., Dubey D., Srivastav N. (2014). Analysis of YouTube as a Source of Information for West Nile Virus Infection. Clin. Med. Res..

[19] https://www.Youtube.Com/Howyoutubeworks/Product-Features/Health-Information/.

[20] Muller A.L., Baker J.F. (2022). Analysis of Lumbar Fusion and Lumbar Arthroplasty Videos on YouTube. Int. J. Spine Surg..

[21] White M.D., Latour K., Giordano M., Taylor T., Agarwal N. (2020). Reliability and Quality of Online Patient Education Videos for Lateral Lumbar Interbody Fusion. J. Neurosurg. Spine.

[22] Stogowski P., Antkowiak L., Trzciński R., Rogalska M., Dułak N.A., Anuszkiewicz K., Kloc W. (2022). Content Quality and Audience Engagement Analysis of Online Videos for Anterior Lumbar Interbody Fusion. World Neurosurg..

[23] Godolias P., Charlot K., Tran A., Plümer J., Cibura C., Daher Z., Dudda M., Schildhauer T.A., Chapman J., Oskouian R.J. (2022). Qualitative Evaluation of Educational Content on Lateral Spine Surgery YouTube^TM^ Demonstrations. Cureus.

[24] Eastwood D., Manson N., Bigney E., Darling M., Richardson E., Paixao R., Underwood T., Ellis K., Abraham E. (2019). Improving Postoperative Patient Reported Benefits and Satisfaction Following Spinal Fusion with a Single Preoperative Education Session. Spine J..

[25] Phan A., Jubril A., Menga E., Mesfin A. (2021). Readability of the Most Commonly Accessed Online Patient Education Materials Pertaining to Surgical Treatments of the Spine. World Neurosurg..

[26] Kocyigit B.F., Nacitarhan V., Koca T.T., Berk E. (2019). YouTube as a Source of Patient Information for Ankylosing Spondylitis Exercises. Clin. Rheumatol..

[27] Charnock D., Shepperd S., Needham G., Gann R. (1999). DISCERN: An Instrument for Judging the Quality of Written Consumer Health Information on Treatment Choices. J. Epidemiol. Community Health.

[28] Bernard A., Langille M., Hughes S., Rose C., Leddin D., Veldhuyzen van Zanten S. (2007). A Systematic Review of Patient Inflammatory Bowel Disease Information Resources on the World Wide Web. Am. J. Gastroenterol..

[29] Koller U., Waldstein W., Schatz K.-D., Windhager R. (2016). YouTube Provides Irrelevant Information for the Diagnosis and Treatment of Hip Arthritis. Int. Orthop..

